# Editorial: A systems approach to personalized exercise and nutrition in health and disease

**DOI:** 10.3389/fspor.2023.1190121

**Published:** 2023-04-12

**Authors:** Diego A. Bonilla, Erika Cione, Fabrizio Angelini, Giuseppe D’Antona, Roberto Cannataro

**Affiliations:** ^1^ Research Division, Dynamical Business and Science Society–DBSS International SAS, Bogotá, Colombia; ^2^ Research Group in Biochemistry and Molecular Biology, Universidad Distrital Francisco José de Caldas, Bogotá, Colombia; ^3^ Research Group in Physical Activity, Sports and Health Sciences (GICAFS), Universidad de Córdoba, Montería, Colombia; ^4^ Department of Pharmacy, Health and Nutritional Sciences, University of Calabria, Rende, Italy; ^5^ Galascreen Laboratories, University of Calabria, Rende, Italy; ^6^ JMedical-Sport Medical Center, Juventus FC, Turin, Italy; ^7^ Centro di Ricerca Interdipartimentale Nelle Attività Motorie e Sportive (CRIAMS)-Sport Medicine Centre, University of Pavia, Voghera, Italy; ^8^ Department of Public Health, Experimental and Forensic Medicine, University of Pavia, Pavia, Italy

**Keywords:** personalized medicine, allostasis and allostatic load, omics, nutraceuticals and functional foods, epigenetic, biological approach

**Editorial on the Research Topic** Systems approach to personalized exercise and nutrition in health and disease

Complex systems are an important field of study that involves several sciences and disciplines to understand how groups of individuals or biological entities interact with one another and their environment. These systems are characterized by their ability to adapt and self-organize, resulting in emergent behaviors that cannot be predicted by studying individual components in isolation. The collective intelligence of these systems arises from the interactions between the individual components and is often greater than the intelligence of any single component. Nonlinearity is a key feature of complex systems, meaning that small changes in initial conditions can lead to large and unpredictable effects over time. As a result, the study of complex systems has important implications for a wide range of fields, including public health, physical activity promotion and noncommunicable disease (NCD) prevention research, policy, and practice (1).

This idea of complexity in biological systems should be addressed from a reformulated insight that studies the fundamental processes of dynamics in living things, from their development (through self-organizing), going through cellular dynamics (structural stability), to physiological dynamics (changes over time). Fractal dimension is particularly useful in the study of complex systems because it allows researchers to quantify and compare the complexity of systems that may be too intricate to analyze using traditional methods. It refers to the degree of complexity or irregularity of a system and is often used to describe patterns that exhibit self-similarity at different scales. In sports sciences, since fractal dimension can provide information on the complexity and irregularity of the time-series data, it can provide insight into the inter- and intracyclic changes of the stroke cycles in swimming (2). Also, fractal dimension has been reported as an index of performance fatigability at the end of a prolonged musculoskeletal contraction, where signal complexity decreases (3). By understanding the fractal dimension of complex systems, researchers can gain insights into their underlying structure, function, and behavior and develop more accurate models to describe and predict their dynamics. Complementarily, systems biology has proposed new visions that have been well-accepted by experimental biology. In this sense, collaborative efforts are in continuous development to have new friendly-user software, bioinformatics, and multi-OMICS tools for practitioners and researchers (4).

Wellbeing and health are dynamical constructs that enclose physical, mental, and social health. As a complex adaptive system, physical and mental health are constantly changing because of internal- or external-level determinants. This intricated set of biological entities and interactions facilitates the occurrence of emergent properties, self-organization, and collective intelligence. In the context of physiological regulation and adaptation, the allostasis–interoception model represents the current paradigm to anticipate stress-mediated needs in health and disease (5). This helps understanding the progression of a disease or a given adaptation process as constantly changing biological situations that are influenced by prior knowledge and the magnitude of the stimuli. Under this model, a chronic stimulus such as energy deprivation, mechanical stress, sleep disturbances, or the rumination process might result in systemic adaptations that reset regulatory parameters to redirect resources toward activities that are more immediately valuable to survival. In this regard, allostatic load has been described as the cost that the system has to pay to reach the new (allostatic) state of adaptation (6).

As any other biological system, the adaptation process in the human being requires two important factors: energy and time. The first has been called recently as the “allostasis and stress-induced energy expenditure” and, therefore, influences total daily energy expenditure. It also might be used to understand the individualized energy restriction for a successful weight loss program, the changes in substrate utilization of a highly trained athlete, the energy dysregulation and altered interoception that are characteristics of the “locked-in” depressed brain, among other phenomena (7, 8). The second factor is related to the restorative period necessary to eliminate interoceptive prediction errors as well as to which different mechanisms will maintain internal states within the bounds of the new setpoint of adaptation. Interestingly, this can be discussed across different levels of biological complexity (organelle, cellular, tissular, physiological, and social). Some of these levels are the time-course alteration in the protein content and enzyme activities as a result of the downstream activation/inhibition of certain signaling pathways that regulate gene expression, the recovery and rest periods between exercise sessions to maintain a certain level of physical performance, or the quality and time of sleep necessary to mentally recover after a stressful day (9). In consequence, both energy- and time-related factors impact the allostatic load of each individual/population. Notwithstanding, in accordance with several scholars, we have reached a point of quasi-consensus that still requires a unified framework of what allostasis–interoception is or does (10).

The research topic “Systems approach to personalized exercise and nutrition in health and disease” opened the door for a comprehensive framework for designing and implementing personalized exercise and nutrition plans that consider an individual's unique biological, behavioral, and environmental factors. This approach recognizes that health and disease are the result of complex interactions among various systems in the body, including the cardiovascular, metabolic, nervous, and musculoskeletal systems, as well as external factors such as diet, lifestyle, and environment. In the mini review “Strength training in elderly: An useful tool against sarcopenia,” the multifactorial etiology of this muscle-wasting condition and the benefits of different resistance training methods are reported. By understanding these interactions and tailoring exercise and nutrition interventions to an individual's specific needs, the systems approach can help optimize health outcomes, prevent and manage chronic diseases, and improve the overall quality of life. In this sense, self-monitoring nutrition and exercise using mobile applications still require improvements for gathering evidence-based information and for validation, as reported in the article “Quality assessment of pre- and postnatal nutrition and exercise mobile applications in the United States and China.” The nutritional approach should also be underlined, particularly in modulating the inflammatory state, both to promote recovery from physical activity and to design a healthy living program; the nutraceutical approach could be highly interesting as evidenced by the Antioxidant, anti-inflammatory and immunomodulatory effects of Spirulina in exercise and sport: A systematic review.

In conclusion, the systems approach has gained increasing attention and recognition in recent years as the prevalence of NCD such as obesity, diabetes, and cardiovascular disease continues to rise and as personalized medicine and healthcare become increasingly important. In the article “Relationship between leisure-time physical activity and depressive symptoms under different levels of dietary inflammatory index”, You et al. provide guidance for the development of prevention strategies with an increased emphasis on the overall effect of physical activity status and diet inflammation for the prevalence of depression. Thus, we encourage researchers to use the information contained in this work and take a more intuitive, integrative, and allostatic view by considering complex systems, network analysis, and the ever-changing and adaptive responses of biological organisms (*Bio-Logic*) (11). In addition, personalized exercise and nutrition should include a deep study of genetics, epigenetics (miRNA, for example) and omics (11–13). Thus, we encourage researchers to use the information contained in this work and take a more intuitive, integrative and allostatic view by considering complex systems, network analysis, and the ever-changing and adaptive responses of biological organisms (Bio-Logic) (14).

## Author contributions

DAB and RC drafted the manuscript. EC, FA, and GD’A commented and revised the draft. All authors contributed to the article and approved the submitted version.
